# FGL-GAN: Global-Local Mask Generative Adversarial Network for Flame Image Composition

**DOI:** 10.3390/s22176332

**Published:** 2022-08-23

**Authors:** Kui Qin, Xinguo Hou, Zhengjun Yan, Feng Zhou, Leping Bu

**Affiliations:** School of Electrical Engineering, Naval University of Engineering, Wuhan 430033, China

**Keywords:** composite flame image, generative adversarial networks, Global-Local, fire mask, data augmentation

## Abstract

It is important to reduce the danger of collecting flame image data sets by compositing flame images by computer. In this paper, a Global-Local mask Generative Adversarial Network (FGL-GAN) is proposed to address the current status of low quality composite flame images. First, FGL-GAN adopts a hierarchical Global-Local generator structure, to locally render high-quality flame halo and reflection, while also maintaining a consistent global style. Second, FGL-GAN incorporates the fire mask as part of the input of the generation module, which improves the rendering quality of flame halo and reflection. A new data augmentation technique for flame image compositing is used in the network training process to reconstruct the background and reduce the influence of distractors on the network. Finally, FGL-GAN introduces the idea of contrastive learning to speed up network fitting and reduce blurriness in composite images. Comparative experiments show that the images composited by FGL-GAN have achieved better performance in qualitative and quantitative evaluation than mainstream GAN. Ablation study shows the effectiveness of the hierarchical Global-Local generator structure, fire mask, data augmentation, and MONCE loss of FGL-GAN. Therefore, a large number of new flame images can be composited by FGL-GAN, which can provide extensive test data for fire detection equipment, based on deep learning algorithms.

## 1. Introduction

In recent years, with the rapid development of artificial intelligence, fire detection algorithms [1,2] based on computer vision have been widely used. However, due to safety concerns, ignition experiments cannot be performed in many indoor environments, such as warehouses, hangars, oil depots, etc. This makes it impossible for fire detection algorithms to test fire images in these scenarios. If the flame (foreground) collected in laboratories can be combined with the above-mentioned indoor environments (background), the above issue can be well addressed.

**Figure 1:**
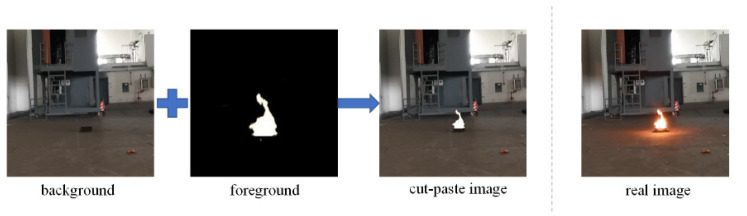
Schematic diagram of cut-paste algorithm.

The simplest image compositing method is to extract the flame from the real image and paste it into the background (cut-paste algorithm called for short, as shown in Figure 1). If the flame image composited by the cut-paste algorithm is compared with the real image, it is evident that there is a marked difference between the composited image and the real image. This is mainly due to the following two reasons. First, there is a halo around the real flame, therefore the flame can transition smoothly into the background and there is no obvious boundary. The cut-paste algorithm cannot paste the halo around the flame in the new background, which renders the composite flame different in appearance from the real flame. Second, the real flame itself is a light source, which will cast reflection on the ground. However, the flame composited by the cut-paste algorithm cannot produce reflection, which makes the composite image unrealistic.

Researchers have made considerable effort in order to address the above problems, which is mainly divided into two categories: traditional image processing and deep learning.

Among traditional methods, the most popular method is to force gradient domain smoothing of the foreground [3,4,5], the most classic of which is Poisson image editing [6]. It maintains the gradient domain between the foreground and the background by computing the gradient of the background and passing it on to the foreground. Traditional methods change the flame’s color and texture information, based on the background information. However, the flame is used as the light source, whose color and texture are not affected by the background during the compositing process. Therefore, traditional methods are not suitable for solving the problem of flame image compositing.

In deep learning methods, image compositing can be considered as an image translation problem, in which the cut-paste composite image is mapped to the real image. However, image translation often makes random changes to the style and content of the background or objects. Therefore, if the image translation algorithm is simply applied to flame image compositing, it can easily cause flame or background distortion.

At present, there is little research on the problem of flame image compositing. Yang et al. proposed FIS-GAN [7]. This network has two independent GANs. The first GAN controls the texture of the flame when the flame is generated by adding code to the input noise. The second GAN reduces blurring of the composite image by adding an attention mechanism to the generator in flame image compositing. However, there are still three problems with this method. First, FIS-GAN is mainly aimed at 128 × 128 image compositing with low resolution and little background information, which will lead to an unsatisfactory fusion effect between background and foreground. Second, FIS-GAN can only generate flame reflection with a fixed area. Under normal circumstances, the area of flame reflection changes with the color, shape of the flame, and background light intensity. The fixed area flame reflection limits the use of FIS-GAN. Finally, the realism of the flame halo and reflection generated by FIS-GAN needs to be improved.

In order to overcome the shortcomings of the above methods, we propose a novel flame image compositing algorithm: FGL-GAN. The network aims to achieve realistic flame image compositing. FGL-GAN uses generative adversarial ideas to learn from the real flame images to composite new flame images. The main contributions of this paper can be summarized into the following four aspects:

1. For the problem of flame image compositing, we propose a new generative adversarial network ‘FGL-GAN’. The generator structure of FGL-GAN is divided into two parts: local generation and global coordination. The local generation part is responsible for generating flame halo and reflection. The global coordination part is responsible for fusing the generated local image with the background. The two parts cooperate with each other to jointly optimize the quality of the composite image;
2. To improve the performance of FGL-GAN, the fire mask and a new data augmentation are proposed in this paper. The fire mask extracted from the original image becomes part of the input to the generation module in FGL-GAN, which can pass information such as the position and shape of the flame to the network, thereby improving the quality of the rendering of flame halo and reflection. Some images of non-flame scenes are also used as training sets for FGL-GAN, to achieve reconstruction of different scene backgrounds and reduce the influence of distractors. At the same time, FGL-GAN introduces the idea of contrastive learning and uses the Modulated Noise Contrastive Estimation (MONCE) loss [8] as part of the overall loss;
3. Extensive comparative experiments and ablation study demonstrate that FGL-GAN achieves the best results for flame image compositing. FGL-GAN has better effectiveness and wider applicability than other networks, and can provide lots of flame datasets for flame detection.

## 2. Related Works

This paper mainly incorporates the method of image translation to study the problem of image compositing. Therefore, this section mainly introduces the research status of image translation and image compositing.

### 2.1. Image Translation

GAN proposed in [9,10,11] makes pixel-level image translation [12,13,14] possible, and has been widely used in image style transfer [15,16], image restoration [17,18], image segmentation [19,20], super-resolution [21,22] and other fields. GAN consists of a generator and a discriminator. The generator generates a fake image, and the discriminator is used to distinguish the generated image from the real image. The generated image is constantly approaching the real image through the adversarial learning between them.

Later, most of the image translation networks adopted the framework of generative adversarial networks. Isola et al. proposed pix2pix [23], which for the first time used conditional generative adversarial networks as a general solution for supervised image translation. But the network’s datasets must be matched. CycleGAN [24] achieved image translation for non-matching datasets by combining G:X (source domain) → Y (target domain) and F:Y → X mappings, and introducing a cycle consistency loss function. However, the network has two mappings, resulting in increasing the amount of network parameters and training time. The above two networks can only achieve one-to-one image translation. In order to achieve translation between multiple datasets, starGAN [25] uses different mask vectors for different datasets. HistoGAN [26] achieves controllable image translation by encoding the color histogram of the input image and feeding it into the network. To improve the resolution of the translated image, Ting-Chun et al. proposed multi-scale generator and discriminator [27], which achieves 2048 × 1024 resolution of the translated image. The translated images of pix2pix and cycleGAN are relatively blurry. Park et al. believed that the general normalization layer loses part of the image information; as a result, they proposed a spatially-adaptive normalization layer [28] to generate realistic images. Hu et al. designed a QS-atten module [29] to improve the quality of the generated images, without changing the model parameters.

However, the above methods basically deal with image translation of public datasets, and do not perform well in solving the problem of flame image compositing. Therefore, this paper proposes FGL-GAN for flame image compositing.

### 2.2. Image Compositing

Image compositing [30,31,32] includes, but is not limited to, the following four issues: object placement, image blending, image harmonization, and shadow generation. Object placement aims to paste the foreground into the background at a reasonable position and size. Refs. [33,34] tries to find a suitable position for the foreground via learning the prior knowledge of the background. Refs. [35,36] predicts the position for the foreground via learning the relationship between the foreground and the background. Image blending aims to refine the foreground boundary and reduce blurriness. The simplest approach is alpha blending [37]. Ref. [38] achieves a smooth transition on the boundary by using the gradient domain constraint as a loss function, according to the Poisson equation, Ref. [39] blends the foreground and background into a seamless new image by using two independent encoders to extract multi-scale characteristics from both. Image harmonization aims to unify the color and lighting characteristics of the foreground and background. Refs. [40,41] count the appearance features of foreground and background (such as color histogram) to unify them through linear or nonlinear transformation. Ref. [42] unifies their appearance by learning the appearance features of foreground and background through an additional spatial separation attention module. Ref. [43] makes the foreground and background consistent by learning the geometry, color, and boundary features between them. Shadow generation aims to generate shadows for foreground objects based on the lighting information of the background, to make the composite image look more realistic. Ref. [44] proposes a lighting inference algorithm that can recover the complete lighting information of the scene from a single image. Ref. [45] designs an interactive software which can generate corresponding soft shadows under user control, according to the lighting information of the background.

Of the four steps of flame image compositing, since manual placement of flame position is more reasonable compared with machine placement, manual placement is chosen as the object placement in this paper. The flame itself is a light source and does not need to be harmonized by the background. Therefore, the flame image compositing can be simplified into two steps: image blending and shadow generation. Image blending is mainly the blurring transition of the boundary between the flame and the background, and the generation of halo around the flame. Shadow generation is mainly the generation of flame reflection. These two issues can be addressed with the image translation model. Therefore, this paper builds an image translation model, FGL-GAN, to solve these two problems together.

## 3. The Proposed Model: FGL-GAN

This section introduces the FGL-GAN proposed in this paper in detail, in four parts: network structure, mask, data augmentation, and loss.

### 3.1. Network Structure

**Figure 2:**
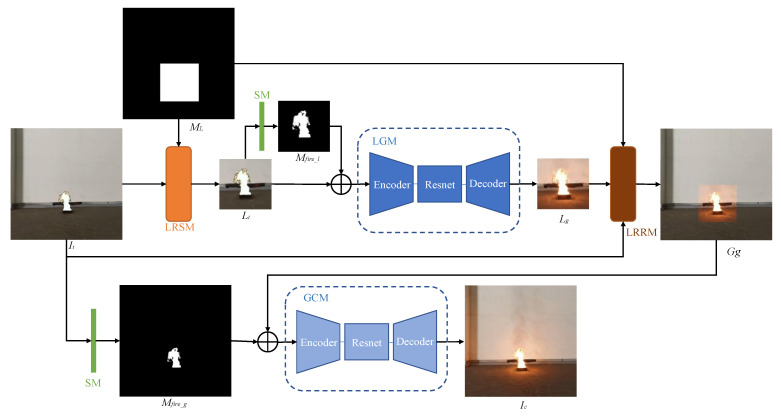
The generator structure.

**Figure 3:**
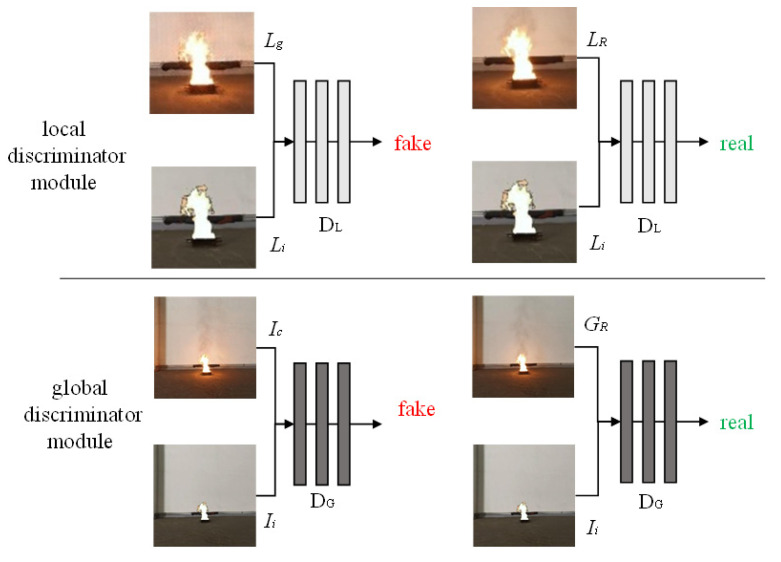
The discriminator structure.

FGL-GAN generates realistic flame halo and reflection through adversarial learning between generator and discriminator. The generator structure of the network is shown in Figure 2. The discriminator structure of the network is shown in Figure 3. The generator and discriminator are introduced in detail below.

#### 3.1.1. Generator

The generator is a Global-Local structure, whose learning goal is to composite realistic flame images. It contains two generation modules: local generation module (LGM) and global coordination module (GCM). LGM aims to make the network focus on local information (around the flame), thereby generating clearer, more realistic, and more detailed flame halo, and reflection. However, if the generator only contains LGM, the color of the generated local image and the background will be inconsistent, and the boundary between the local image and the background will be too sharp, resulting in an unnatural and unrealistic overall image. Therefore, the generator blends the local image with the background color and blurs the boundaries through GCM, so that the final composite image is more natural and realistic. The two generation modules are coordinated and optimized together to composite the final flame image.

The generator contains two local operation modules: local region section module (LRSM) and local region recovery module (LRRM). The generator selects the local region to be composited through the LRSM as the input of the LGM. The generator maps the local image back to the overall image through LRRM as input to GCM. The generator uses LRSM and LRRM to separate flame halo and reflection generation and global coordination into two parts, thus reducing the difficulty of each part.

The generator also contains a segmentation module (SM) designed to implement flame image segmentation. SM uses the segmented local fire mask (*M_fire_l_*) and global fire mask (*M_fire_g_*) as the input of LGM and GCM, respectively. The two fire masks enable LGM and GCM to focus on flame and its surroundings, instead of image style transfer, thereby improving the quality of composite images.

The steps of image compositing inside the generator are: *I_i_* is obtained through cut-paste algorithm (as shown in Figure 1). The generator receives *I_i_* and local mask *M_L_* obtained by the cut-paste algorithm. First, the local image *L_i_* is selected from *I_i_* by LRSM. The local fire mask *M_fire_l_* is segmented from *L_i_* by SM. Then *L_i_* combined with *M_fire_l_* are inputted into LGM to render flame halo and reflection. LGM outputs local composite image *L_g_*. *L_g_*, *I_i_*, and *M_L_* are composited into global image *G_g_* by LRRM. Finally, global fire mask *M_fire_g_* is obtained by *I_i_* through SM. *M_fire_g_* combined with *G_g_* and are inputted into GCM for global coordination. GCM outputs the final composite flame image *I_c_*.

#### 3.1.2. Discriminator

The goal of the discriminator is to distinguish the authenticity of the composite image. It contains two sub discrimination modules: local discriminator module (*D_L_*) and global discriminator module (*D_G_*). When *D_L_* has *L_g_* as input, the output is probability that *L_g_* is false. When *D_L_* has *L_R_* as input, the output is probability that *L_R_* is real. In order to make the output *L_g_* of LGM consistent with the input *L_i_* in texture, *L_g_* and *L_i_* or *L_R_* and *L_i_* are combined as the input to *D_L_*. *D_G_* is similar to *D_L_*, except that the inputs are replaced with global *I_c_* and *G_R_*.

### 3.2. Mask

Compared with the general image translation network, in order to make the generator focus on changing the perimeter of the flame rather than the overall style of the image, this paper designed two masks in FGL-GAN: fire mask and local mask.

Fire mask contains *M_fire_l_* and *M_fire_g_*. They are obtained by segmenting the flame from local or global images, and are used as part of the inputs to the two generation modules. Because the generation modules do not want to change the color and texture of the flame, the generator inputs the fire mask to the generation module and directly conveys the position, shape, size, and other information of the flame, to the generation module. This makes the generation module focus on the flame halo and reflection generation. Fire mask can avoid the occurrence of flame shape deformation, color distortion, and the change of overall style of the image in the process of flame image compositing.

Local mask (*M_L_*) is artificially set. LRSM takes *M_L_* as the criterion to select a specific local region *L_i_* from the initial *I_i_* according to Equation (1). LRRM takes *M_L_* as the criterion, and combines the flame local image *L_g_* generated by LGM, and the initial *I_i_*, into a new global image *G_g_* according to Equation (2). *M_L_* is the valve of LRSM and LRRM. Users can control *M_L_* to change the flame position and region of halo and reflection. The size of the local mask should not be fixed. The size of the local mask is artificially set and can affect the area of the flame reflection.

(1) $$L_{i}\left(x,y\right)=\{\begin{array}{cc} I_{i}\left(x,y\right) & M_{L}\left(x,y\right)=1\\ NaN & M_{L}\left(x,y\right)=0 \end{array}$$

where (*x*, *y*) is the pixel of the image.

(2) $$G_{g}\left(x,y\right)=\{\begin{array}{cc} L_{g}\left(x,y\right) & M_{L}\left(x,y\right)=1\\ I_{i}\left(x,y\right) & M_{L}\left(x,y\right)=0 \end{array}$$

### 3.3. Data Augmentation

The reasons why data augmentation technology is used in this paper are as follows: First, the flame datasets used in this paper have only 24 different scenes. When there are fewer scenes, the network is prone to overfitting, resulting in blurred or even distorted background of the composite images. Second, the network should learn to recognize flames. It can render flame halo and reflection in scenes with fire, and only completes the background reconstruction in flameless scenes. However, when there are fewer scenes, it is difficult for the network to complete the background reconstruction in flameless scenes. Finally, when there are fewer training scenes, the network is easily affected by distractors (objects similar to flames, such as strong lights, car lights, flashlights), and is prone to generate halo and reflection around the distractors. Therefore, data augmentation is necessary.

**Figure 4:**
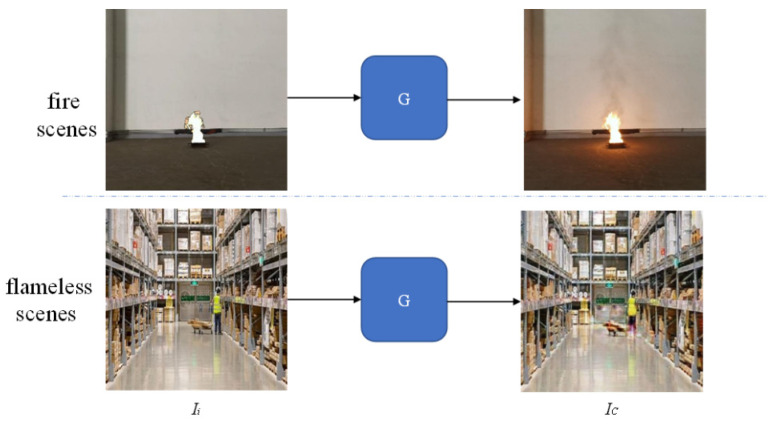
Data augmentation.

In order to avoid the above problems, this paper uses a large number of flameless scenes as part of the network training set. The purpose is so that the network not only learns the compositing of flame images in scenes with flame, but also learns the reconstruction of the background in the flameless scenes (as shown in Figure 4, the size and location of the local mask is a random setting when the input image is a flameless image). The two goals are not in conflict because, in the fire scenes, except in regions around the fire, most of the other background regions should also be reconstructed. Therefore, the data augmentation in this paper is to input the flameless scenes into the network with a certain probability, as shown in the following equation:

(3) $$I_{i}=\alpha I_{fire}+\left(1-\alpha \right)I_{no\_fire}$$

where α is the proportional coefficient for adjusting the flame scenes and the flameless scenes.

This data augmentation increases the richness of the data, which can suppress the network from overfitting and reduce the blurriness of the composite image. In addition, this data augmentation will also improve the network’s ability to identify flames, which can enable the network to complete background reconstruction in flameless scenes. Finally, the increase of training samples will make it easier for the network to learn the characteristics of flames and distractors, so that the impact of distractors on the network will also be reduced.

### 3.4. Loss

In order to better solve the problem of flame image compositing, this paper designed four sub losses, including: local adversarial loss, local L1 loss [23], global adversarial loss, and MONCE loss [8].

#### 3.4.1. Local Adversarial

Adversarial loss is common in GANs [46,47,48]. Its purpose is to make the generator and discriminator learn adversarially. The local discriminator strives to distinguish the local image, *L_g_*, generated by the LGM in the generator, from the real local image, *L_R_*, but the goal of LGM is to render flame reflection and halo as realistic as possible. Local adversarial loss is defined as:

(4) $$L_{GAN}^{L}\left(G,D_{L}\right)=E_{L_{i},L_{R}}\left[\log\;D_{L}\left(L_{i},L_{R}\right)\right]+E_{L_{i}}\left[1-\log D_{L}\left(L_{i},L_{g}\right)\right]$$

#### 3.4.2. Local L1 Loss

In order to ensure the similarity between the generated local image, *L_g_*, and the real local image, *L_R_*, this paper uses *L*1 regularization to calculate the error between the generated image and the real image, pixel by pixel. Local *L*1 loss is defined as:

(5) $$L_{L1}^{L}\left(G\right)=∥L_{R}-L_{g}{∥}_{1}$$

#### 3.4.3. Global Adversarial Loss

The global discriminator strives to distinguish global image, *I_c_*, composited by the generator, from the real global image, *G_R_*, while the goal of the generator is to make the composite flame images as consistent as possible with the real flame images. Global adversarial loss is defined as:

(6) $$L_{GAN}^{G}\left(G,D_{L}\right)=E_{I_{i},G_{R}}\left[\log D_{G}\left(I_{i},G_{R}\right)\right]+E_{I_{i}}\left[1-\log D_{G}\left(I_{i},I_{c}\right)\right]$$

#### 3.4.4. MONCE loss

In order to reduce the blurriness of composite images, this paper introduces contrastive losses [49,50,51]. However, the general contrastive losses tend to set all negative samples to the same weight, which will reduce the performance of the generator. However, MONCE designates a negative sample similarity target matrix to retrieve an optimal negative sample weight through optimal transport [52], which can improve the quality of the composite images. This paper adopts the MONCE loss and it is defined as:

(7) $$L_{MONE}\left(X,Y\right)=-\overset{N}{\underset{i=1}{\sum }}\log\frac{e^{x_{i}\cdot y_{i}/\tau }}{e^{x_{i}\cdot y_{i}/\tau }+Q\left(N-1\right)\sum_{\begin{array}{c} j=1\\ j\neq i \end{array}}^{N}w_{ij}\cdot e^{x_{i}\cdot y_{i}/\tau }}$$

where *X =* [*x*_1_, *x*_2_, …, *x_N_*] are image feature sets encoded from composite images *I_c_*. *Y =* [*y*_1_, *y*_2_, …, *y_N_*] are image feature sets encoded from real images *G_R_*. *τ* is the temperature parameter. *N* is the number of feature patches. *Q* is the weight of negative terms. *w_ij_* is the coefficient obtained by optimal transport (as shown in Equation (8).

(8) $$\min_{w_{ij},i,j}\left[\overset{N}{\underset{i=1}{\sum }}\overset{N}{\underset{\begin{array}{c} j=1\\ j\neq i \end{array}}{\sum }}w_{ij}\cdot e^{x_{i}\cdot y_{i}/\beta }\right]\;s.t.\overset{N}{\underset{i=1}{\sum }}w_{ij}=1,\overset{N}{\underset{j=1}{\sum }}w_{ij}=1$$

where *β* is the temperature parameter of the cost matrix in optimal transport.

The overall loss is the sum of the above losses and is defined as:

(9) $$L_{total}\left(G,D_{L},D_{G}\right)=L_{GAN}^{L}+\lambda_{1}L_{GAN}^{G}+\lambda_{2}L_{L1}^{L}+\lambda_{3}L_{MONCE}$$

where *λ*_1_, *λ*_2_, *λ*_3_ are the weights for adjusting each loss.

## 4. Implementation

This section introduces the implementation details of the network from the following three aspects: datasets production, generator structure, and discriminator structure.

### 4.1. Datasets Production

At present, there is no common datasets for flame image composite. To produce the datasets, we took 24 fire videos of different scenes through experiments, converted them into 5000 images, and divided them into training sets, validation sets, and test sets by 8:1:1 ratio. Furthermore, we searched the Internet for some images of warehouses and hangars (without fire) as part of the training sets.

**Figure 5:**
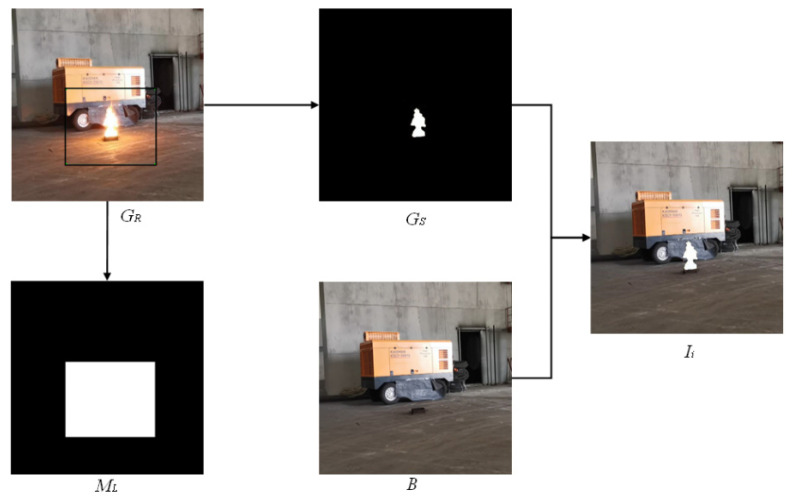
Datasets production process.

The production process of the datasets is shown in Figure 5. Details are as follows: First, local mask is obtained by artificially marking the flame and the area of its halo, and reflection. Second, the flame is segmented from the original image. Then, the flame is pasted directly to the background image according to Equation (10). Finally, the flame, flame halo, and reflection, are labeled from the original image to form a local mask *M_L_*.

(10) $$I_{i}=G_{s}\cdot M_{fire\_g}+B\cdot \left[1-M_{fire\_g}\right]$$

where *G_s_* is flame which is segmented from the original image. *B* is the background. *M_fire_g_* is the fire mask. *I_i_* is the image where the flame is pasted directly to the background.

### 4.2. Generator Structure

LRSM and LRRM have been introduced above and their details will not be repeated here. This subsection mainly introduces the two generation modules and the segmentation modules.

#### 4.2.1. Generation Modules

**Figure 6:**
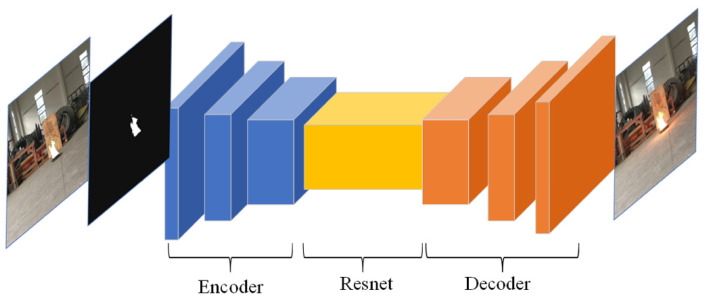
The generation module structure.

The two generation modules are the classic Encoder-Resnet-Decoder structure (as shown in Figure 6). Encoder-Decoder is a symmetrical structure, which ensures the consistency of input and output image sizes. Resnet network [53] introduces residual error, which improves the learning ability of the network. The two generation modules are only different in the number of layers of the resnet block. Due to the small size of local images, the LGM has seven layers, but the GGM has 10 layers. The detailed structure of each block in the generator is Appendix A.

#### 4.2.2. Segmentation Modules

With reference to [54,55], the obtained flame images have the following unique color characteristics, compared with images from other objects: (1). *R* component value of the flame is higher than the other component values. (2). The *B* component value and *B* component dispersion of the flame and the distractors are quite different. (3). The S component of the flame is linearly related to the *R* component. Using the above color characteristics of the flame images, the criterion for the flame segmentation module designed in this paper is shown in Equation (11). Equation (11) can retain flame information, while suppressing interferences such as flame reflection and sunlight, with good segmentation effect.

(11) $$f\left(x,y\right)=\{\begin{array}{lll} f\left(x,y\right) & & R\geq G\geq B\&B\geq B_{T}\\ & & \&S>\left(1-R\right)S_{T}/R_{T}\&B_{std}\geq B_{std\_T}\\ 0 & & else \end{array}$$

where f(x, y) is the color value at each position in the image *R*, *G*, *B*, *S* are the red, green, blue, and saturation components of the image, respectively. *B_T_*, *R_T_*, *S_T_* are blue component threshold, red component threshold, and saturation component threshold, respectively. *B_std_* is the blue component dispersion of a certain area in the image [55]. *B_std_T_* is blue component dispersion threshold.

### 4.3. Discriminator Modules

**Figure 7:**
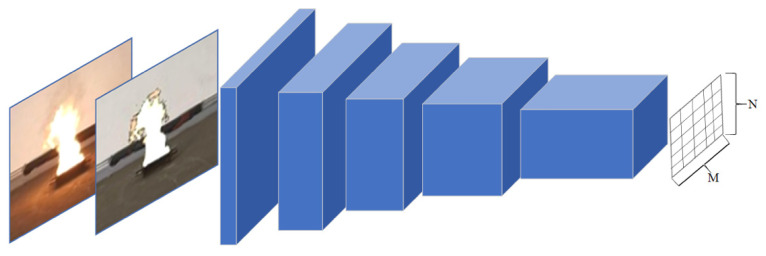
The local discriminator module structure.

The local discriminator module adopts the structure of PatchGAN (as shown in Figure 7). PatchGAN has no limit on the size of input images and outputs an M × N matrix. Each element in the matrix represents the authenticity of a patch in the original image, therefore the final target value is the average of all elements in the matrix. The global discriminator adopts the structure of DCGAN [56] and outputs the probability of the authenticity of the global image (The detailed structure of the global discriminator is Appendix A).

## 5. Experiment Results

The FGL-GAN designed in this paper, along with current mainstream image translation networks including pix2pix [23], cycleGAN [24], QS-attn [29], and FIS-GAN [7], which is the only generative adversarial network for flame image compositing, was iterated 400,000 times under the same conditions on RTX2080Ti. The results are evaluated and compared qualitatively and quantitatively.

### 5.1. Quantitative Evaluation Metrics

The quantitative evaluation in this paper is gauged in three aspects: the comparison between composite images and real images, computer vision, and user evaluation.

*FID* is selected for the comparison between composite images and real images. *FID* refers to the process in which the composite images are inputted into the trained inception [57] network, and Fréchet Inception Distance [58] is calculated with features extracted from the layer before the last fully connected layer. *FID* can evaluate the similarity between composite images and real images, and the clarity and diversity of composite images. The lower the *FID* value, the better the effect of image compositing.

Resnet accuracy (acc) [53] and yolov5 [59] confidence(conf) were selected for computer vision. Resnet accuracy refers to the classification accuracy of fire and non-fire on the composite images, from the same trained resnet network. The higher the classification accuracy, the better the authenticity of the composite images. Yolov5 confidence refers to the probability that a certain object in the composite image is determined to be fire, from a trained yolov5 network. The higher the yolov5 confidence, the more realistic the composite image, and the better the rendering of flame halo and reflection.

User evaluation refers to the process in which 10 users select one with the best composite effect, from a group of images composited by all networks. The score for one network is the ratio of the best-performing images composited by that network, to the total number of composite images. The evaluation process is divided into global and local. From the global perspective, users mainly compare the authenticity of the image composition. From the local perspective, users mainly compare the generation effects of flame halo and reflection.

### 5.2. Qualitative Evaluation

The qualitative evaluation in this paper was carried out on test sets and prediction sets. The real images of the test sets were known, the background and flame position were the same as the training sets, but the flame was different to the training sets. The real images of the prediction sets were unknown, and the background, flame, and flame position, were all different to the training sets.

**Figure 8:**
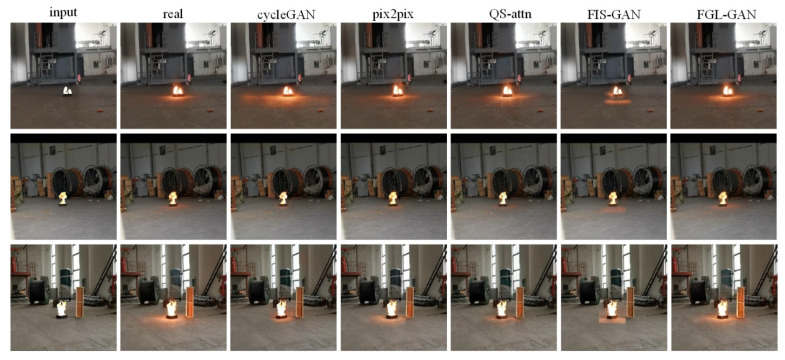
The example of the test sets.

One example of the test sets is shown in Figure 8. In Figure 8, it can be observed that the reflective area rendered by the cycleGAN composite images is randomly larger, or smaller, than the real images. The flame halo and reflection rendered by the QS-attn composite images are relatively blurry, and are quite different from the real images. The composite images by FIS-GAN have a clear boundary between the flame reflection and the background, and is relatively blurry. Although the flame halo and reflection rendered by pix2pix and FGL-GAN are more reasonable, the halo and reflection rendered by FGL-GAN are closer to the real images, than pix2pix.

**Figure 9:**
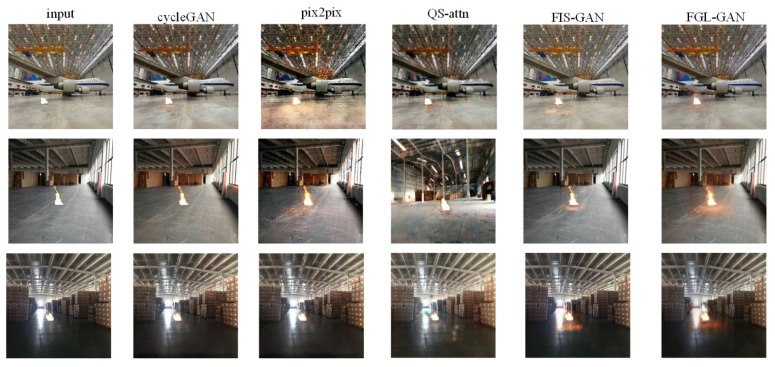
An example of the prediction sets.

One example of the prediction sets is shown in Figure 9. The background light of the example images range from light to dark. In Figure 9, it can be observed that cycleGAN can composite more realistic images when the light is bright, but can only generate halo, and basically cannot render reasonable reflection when the light is moderate or dark. Pix2pix can composite more realistic images when the light is moderate. However, the images composited by pix2pix are distorted (the entire ground becomes a flame reflective area) when the light is bright. Additionally, pix2pix cannot render reasonable reflection when the light is dark. The overall image style of composite images rendered by QS-attn is easily altered, which makes the images unreal. Also, flame halo and reflection rendered by QS-attn are poor among all the networks. There is a clear boundary between local image composited by FIS-GAN and the background, which makes the composite images look unreal. Flame halo and reflection rendered by FIS-GAN are unreasonable: flame halo rendered by FIS-GAN is poor, which makes the border between the flame and the background too sharp. Normal flame reflection should spread from the bottom of the flame towards the ground, and diminish gradually. However, the flame reflection rendered by FIS-GAN always clusters under the flame, and its aberration is not obvious.

From a global perspective, the boundary between flame and background in the images composited by FGL-GAN is smooth, its background style will not change, it will not distort, and it is more natural and realistic. From a local perspective, the flame halo and reflection rendered by FGL-GAN are more reasonable. First, the flame halo and reflection change with the background light. When the light is strong and natural light is the main light source, the reflection of the flame is not obvious; as the light weakens, the flame gradually becomes the main light source and the reflection becomes more and more obvious. Second, the rendered reflection of the flame gradually attenuates with increasing distance from the flame. Finally, the rendered flame halo and reflections are less affected by distractors. Compared with other networks, the FGL-GAN proposed in this paper has the best effect on composited flame images.

### 5.3. Quantitative Evaluation

This paper tested the *FID*, resnet accuracy, yolov5 confidence, and global and local user evaluation, composited by each network. The results are shown in Table 1. The *FID* comparison results show that compared with other networks, the FGL-GAN proposed in this paper has the lowest *FID* of 29.75, which indicates that the halo and reflection rendered by FGL-GAN are closest to the real images, and the clarity and diversity of the composite images are also the best. From the perspective of computer vision algorithm evaluation, resnet accuracy, and the yolov5 confidence of FGL-GAN, achieve 0.9386 and 0.7534, respectively, which are both better than other networks. It shows that the flame halo and reflection composited by FGL-GAN can “deceive” the fire classification and recognition algorithms, based to certain extent on computer vision, and can achieve the purpose of “mixing the fake with the real”. From the perspective of user evaluation, compared with other networks, users incline to choose the images composited by FGL-GAN, whether for the authenticity of the image as a whole or for local rendering of the flame halo and reflection. This shows the images composited by FGL-GAN are more in line with the aesthetics of human vision, and are easier to deceive humans. To sum up, the quality of the flame images composited by FGL-GAN proposed in this paper excels, compared to the other methods.

### 5.4. Ablation Study

We conducted ablation study to evaluate the effectiveness of each module of the method proposed in this paper, which can be divided into five cases: only LGM, only GCM, no MONCE loss, no fire mask, and FGL-GAN.

**Figure 10:**
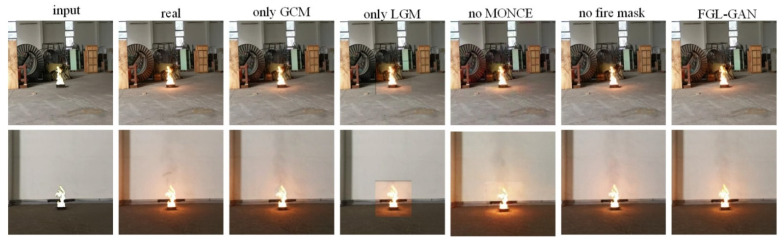
Ablation study of test sets.

**Figure 11:**
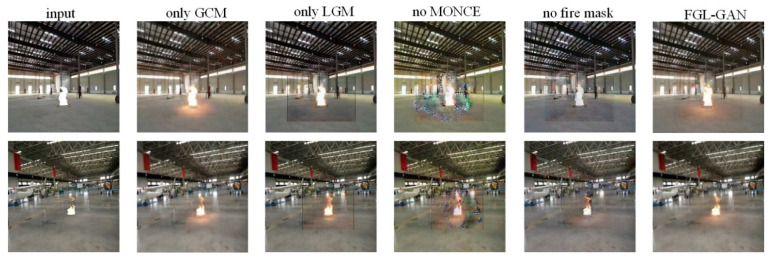
Ablation study of prediction sets.

The example of test sets is shown in Figure 10, and the example of prediction sets is shown in Figure 11. In Figure 10 and Figure 11, compared with only LGM and FGL-GAN, only LGM produced an obvious border between the local composite image and the background, which would cause the overall image to be unreal, while FGL-GAN adds GCM after LGM, which would make the local image and the background blend better and make the images more realistic. Compared with only GCM and FGL-GAN, the flame halo and reflection rendered by only GCM were more blurred than FGL-GAN, which shows that adding the LGM before the GCM can effectively improve the clarity of rendering flame halo and reflection in composite images. Compared with no MONCE loss or FGL-GAN, the composite images without the former loss in the prediction sets were obviously distorted, and there was an obvious boundary between the local image and the background. Therefore, adding the MONCE loss to the network can prevent the composite images from distortion. Compared with no fire mask and FGL-GAN, flame halo and reflection in the prediction sets were basically not rendered with no fire mask. FGL-GAN adds fire mask to the input of the two generation modules of the network, which can provide shape, position, size of the flame, and other information to the network, thereby rendering reasonable flame halo and reflection.

The comparison of *FID* and yolov5 confidence in five cases is shown in Table 2. *FID* comparison results show: *FID* of FGL-GAN is slightly better than no fire mask and with only global coordination module, indicating that adding local generation module and fire mask to the network can improve resemblance between composite images and real images, as well as the clarity of composite images. There is a big gap in *FID* between FGL-GAN and the two cases where there was only LGM and no MONCE loss in the network, indicating that the GCM and MONCE loss in the network can significantly improve the quality of composite images. Yolov5 confidence comparison results show: FGL-GAN (combining with LGM and GCM) has better confidence than a single module, indicating effectiveness of FGL-GAN co-optimizing the two modules. Compared with no MONCE and no fire mask, the confidence of FGL-GAN can be significantly improved. The main reasons are shown in Figure 11: the network with no MONCE loss can cause distorted composite images in the prediction sets. The network with no fire mask can cause significantly reduced effect in halo and reflection rendering.

**Figure 12:**
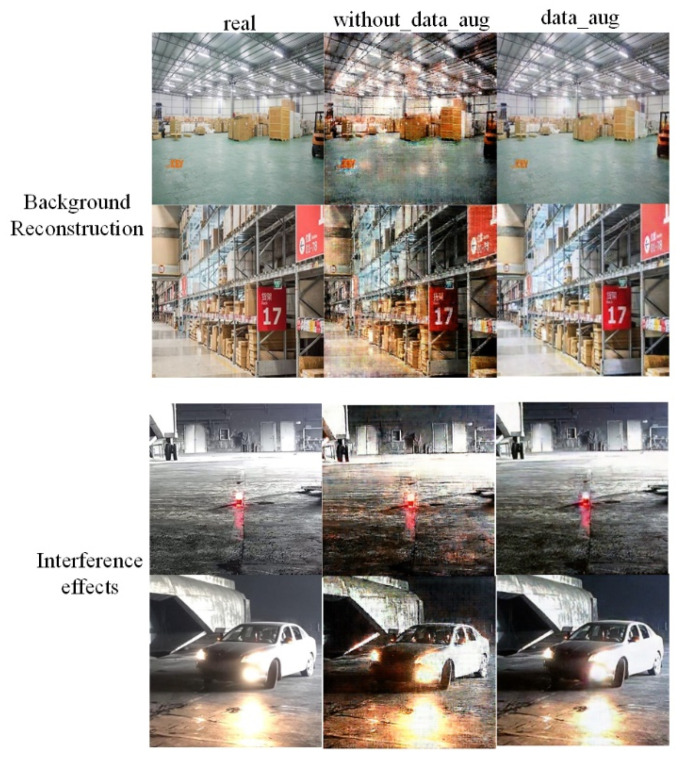
Comparison with before and after, using data augmentation.

This paper explores the background reconstruction and the influence of distractors on the network before and after data augmentation, as shown in Figure 12. Compared with before and after data augmentation, the background reconstruction is relatively blurred, and the network is easily affected by interference, which can cause flame halo and reflection near alarm lights or headlights before data augmentation. However, the use of data augmentation can significantly improve the clarity of background reconstruction and reduce the impact of distractors on the network.

**Figure 13:**
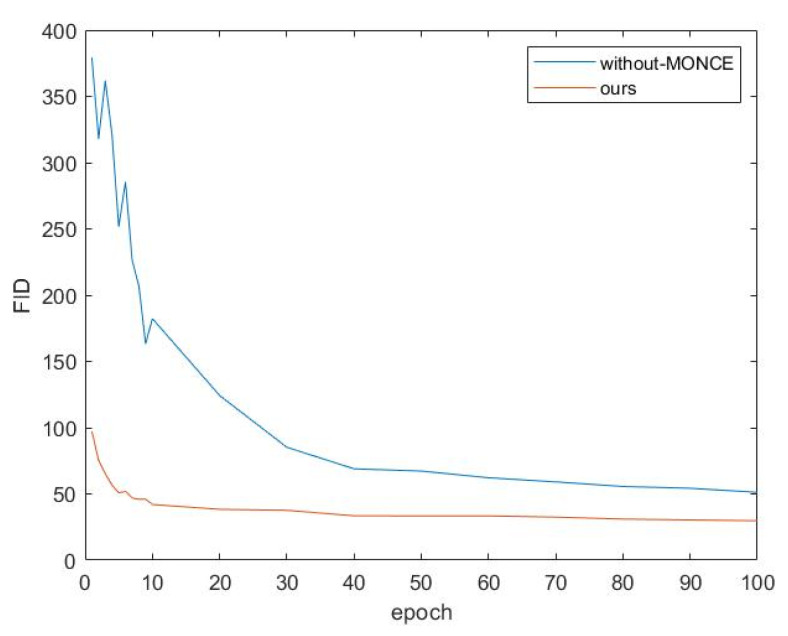
Variation of *FID* with the number of training epochs.

This paper explores the impact of using MONCE loss on network fitting speed, as shown in Figure 13. It can be observed that using MONCE loss can significantly speed up network fitting.

In addition, an experiment was carried out to study the effect of the size of the local mask on the area of flame reflection. Detailed experiments are in Appendix B.

## 6. Conclusions

This paper proposes a novel generative adversarial network, FGL-GAN, for flame image compositing. First, FGL-GAN aims to achieve local and global co-optimization through the Global-Local structure. Second, the fire mask is inputted to the generation module to improve the quality of flame halo and reflection rendering. Then, the use of new data augmentation techniques aims to enhance the immunity of the network to interference. Finally, FGL-GAN introduces MONCE loss to improve the network’s performance.

Qualitative comparative evaluations of FGL-GAN with existing methods show FGL-GAN can composite better flame images from the global and local perspective in different circumstances, compared to other methods. Quantitative comparative evaluations of FGL-GAN with existing methods show the images composited by FGL-GAN reach 29.75 on the *FID*, reach 0.9386 on resnet accuracy, reach 0.7534 on yolov5 confidence, reach 0.583 on global user evaluation, and reach 0.636 on local user evaluation. The results of FGL-GAN are all better than the other compared methods.

A series of ablation experiments demonstrated that the hierarchical Global-Local generator structure, fire mask, and the MONCE loss of FGL-GAN, can significantly improve the quality of composited images. The data augmentation of FGL-GAN improves the anti-interference performance of the network. The MONCE loss can significantly speed up network fitting.

In summary, relatively realistic virtual fire images can be composited by FGL-GAN in places where fire experiments cannot be performed. Therefore, the images composited by FGL-GAN can used to test fire detection algorithms.

In Figure 9, it can be observed that the quality of FGL-GAN composite images when the light is moderate, is better than when light is too bright or too dark. This is mainly because the training sets are mostly images with moderate light. To solve this problem, we will continue to optimize FGL-GAN.

## Figures and Tables

**Table 1:** Quantitative evaluation results.

		cycleGAN	pix2pix	QS-attn	FIS-GAN	FGL-GAN
*FID*		47.10	40.29	59.52	53.25	29.75
Computer vision	acc	0.7778	0.7222	0.7389	0.9115	0.9386
	conf	0.6067	0.5788	0.5928	0.6828	0.7534
User evaluation	global	0.167	0.092	0.125	0.033	0.583
	local	0.027	0.118	0.040	0.179	0.636

**Table 2:** Comparison of *FID* and yolov5 confidence in different situations of ablation study.

	Only GCM	Only LGM	No MONCE	No Fire Mask	FGL-GAN
*FID*	33.08	51.37	51.21	31.40	29.75
conf	0.7332	0.7046	0.6682	0.6915	0.7534

**Figure A1:**
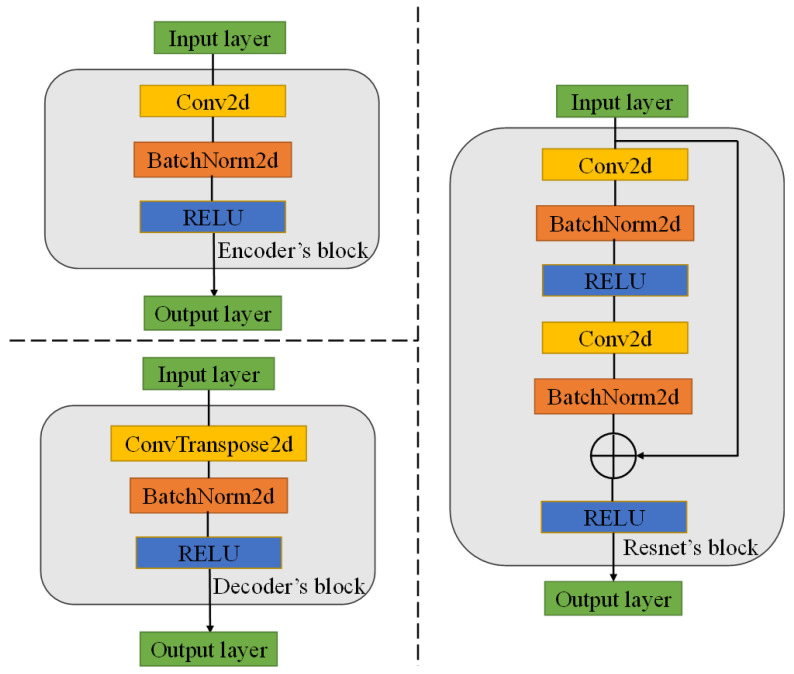
The specific structure of “Encoder-Resnet-Decoder”.

**Figure A2:**
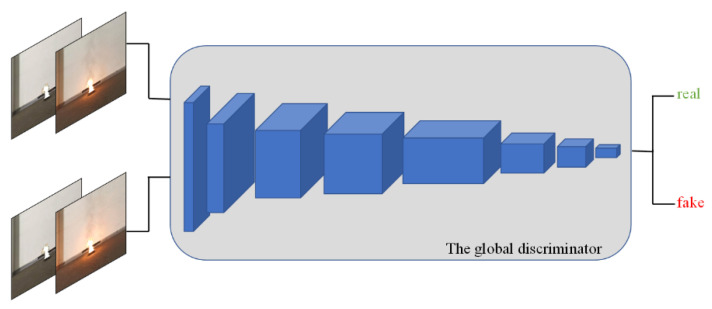
The structure of the global discriminator.

**Figure A3:**
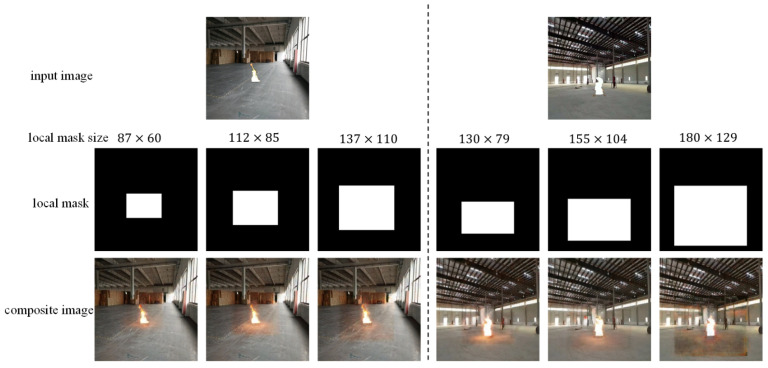
The effect of the size of the local mask on the area of flame reflection.

## Data Availability

Not applicable.

## Appendix A

The generator and discriminator structures in FGL-GAN are described in detail in this appendix:

The LGM and GGM in the generator takes the structure of “Encoder-Resnet-Decoder”. The specific structure of each block is shown in Figure A1.

The structure of the local discriminator is described in detail in the text. Only the structure of the global discriminator is described in Figure A2. Each block in the figure represents “Conv2d-Activate layer-Norm layer”.

## Appendix B

An experiment was carried out to study the effect of the size of the local mask on the area of flame reflection. The input images are combined with different local mask in this experiment. They are all fed into FGL-GAN to composite images. The experimental results are shown in Figure A3. It can be observed that the area of flame reflection will increase with the size of the local mask.
